# Positive Selection of Natural Poly-Reactive B Cells in the Periphery Occurs Independent of Heavy Chain Allelic Inclusion

**DOI:** 10.1371/journal.pone.0125747

**Published:** 2015-05-19

**Authors:** Ying Xing, Qiuhe Ji, Ying Lin, Meng Fu, Jixin Gao, Ping Zhang, Xingbin Hu, Lei Feng, Yufeng Liu, Hua Han, Wei Li

**Affiliations:** 1 Department of Dermatology, Xijing Hospital, Fourth Military Medical University, Xi’an, China; 2 Department of Endocrinology and Metabolism Disease, Xijing Hospital, Fourth Military Medical University, Xi’an, China; 3 State Key Laboratory of Cancer Biology, Department of Medical Genetics and Developmental Biology, Fourth Military Medical University, Xi’an, China; 4 Department of Otolaryngology Head and Neck surgery, Xijing Hospital, Fourth Military Medical University, Xi’an, China; Institut Pasteur, FRANCE

## Abstract

Natural autoreactive B cells are important mediators of autoimmune diseases. Receptor editing is known to play an important role in both central and peripheral B cell tolerance. However, the role of allelic inclusion in the development of natural autoreactive B cells is not clear. Previously, we generated μ chain (TgV_H_3B4I) and μ/κ chains (TgV_H/L_3B4) transgenic mice using transgene derived from the 3B4 hybridoma, which produce poly-reactive natural autoantibodies. In this study, we demonstrate that a considerable population of B cells edited their B cells receptors (BCRs) via light chain or heavy chain allelic inclusion during their development in TgV_H_3B4I mice. Additionally, allelic inclusion occurred more frequently in the periphery and promoted the differentiation of B cells into marginal zone or B-1a cells in TgV_H_3B4I mice. B cells from TgV_H/L_3B4 mice expressing the intact transgenic 3B4 BCR without receptor editing secreted poly-reactive 3B4 antibody. Interestingly, however, B cell that underwent allelic inclusion in TgV_H_3B4I mice also produced poly-reactive autoantibodies in vivo and in vitro. Our findings suggest that receptor editing plays a minor role in the positive selection of B cells expressing natural poly-reactive BCRs, which can be positively selected through heavy chain allelic inclusion to retain their poly-reactivity in the periphery.

## Introduction

The ability of B cells receptor (BCR) variable (V) region gene fragments to rearrange randomly during early B cell development is of great significance. It not only increases the diversity of BCR specificities [1], but also increases the possibility of autoantibody production. It has been suggested that the prevalence of poly-reactive B cells to various autoantigens is more than 50% in early B cells precursors [2]. However, this number is reduced to approximately 5% after B cell maturation. Many studies based on immunoglobulin (Ig) gene transgenic mice have shown that the deletion of autoreactive B cell clones is induced by central tolerance mechanisms, including clonal deletion, anergy and receptor editing [3–7], during B cells development. Among these mechanisms, receptor editing is critical for central B cells tolerance [8], through which autoreactive B cells that are destined for clonal deletion or anergy can be rescued by successful secondary rearrangement of their BCR genes. Receptor editing plays important roles in both positive and negative selection of autoreactive B cells [9], suggesting a relationship between receptor editing and autoimmune diseases [10, 11]. Consistently, the persistence of pathological autoantibodies has been associated with attenuated receptor editing in the bone marrow (BM) or periphery in autoimmune disease mouse models and patients [12–14]. Studies with other models have suggested that significant receptor editing is elicited in the development of autoreactive B cells [15–17]. However, there is no direct evidence showing that defects in receptor editing enhance autoantibody production in autoimmune diseases.

Most of the naturally-occuring autoantibodies are poly-reactive and exist in healthy individuals [18, 19]. Recent studies have suggested that 5~20% of long-lived B cells are autoreactive in humans [2]. However, the role of receptor editing in the development of natural autoreactive B cells is not yet clear. Secondary recombination at the light (L) chain genetic loci generates a new μ chain that can either substitute the autoreactive L chain [20], or can be co-expressed on the cell surface as a “passenger” together with the original L chain, and can also associate with the heavy (H) chain separately. This later phenomenon is referred to as allelic inclusion [21, 22] and is a result of receptor editing. The co-expression of an “innocent” L chain can rescue B cells from negative selection by diluting the surface expression of the self-reactive BCR [23]. In addition to L chains, secondary rearrangement of V genes also happens at the H chain loci [24, 25]. However, the extent and function of H chain allelic inclusion are unknown. Given the dominant role V_H_ plays in antigen recognition, it will be important to clarify the relationship between H chain allelic inclusion and receptor editing in the generation of natural autoreactive B cells, to reveal the mechanisms of B cell tolerance.

We have established μ chain transgenic mice with the V_H_ gene derived from 3B4 hybridoma producing a natural autoantibody [26]. Nine founders were generated with different allelic exclusion efficiency. In the present study, B cells from one founder line (named as TgV_H_3B4I) with apparent allelic inclusion and receptor editing were analyzed. We also generated κ chain transgenic mice (TgV_L_3B4) with the V_L_ gene from the same 3B4 hybridoma and double transgenic mice (TgV_H/L_3B4) were created by breeding TgV_H_3B4 mice and TgV_L_3B4 mice. In contrast to B cells from TgV_H_3B4I mice expressing 3B4 μ chain, we did not observe any significant receptor editing in B cells from TgV_H/L_3B4 mice which expressed the whole 3B4 natural poly-reactive BCR. B cells expressing endogenous self-reactive L chains escaped negative selection by H chain allelic inclusion in the periphery, and these B cells differentiated into special subsets with the ability to secrete poly-reactive antibodies, just like B cells expressing the integrated 3B4 BCR in TgV_H/L_3B4 mice without allelic inclusion. Our findings suggest that B cells with natural poly-reactivity can be positively selected with or without H chain allelic inclusion.

## Materials and Methods

### Ethics Statements

The animal husbandry, experiments and welfare were conducted in accordance with the Detailed Rules for the Administration of Animal Experiments for Medical Research Purposes issued by the Ministry of Health of China, and were approved by the Animal Experiment Administration Committee of Fourth Military Medical University. Mice were raised in the specific pathogen free conditions on the C57BL/6 background, and were manipulated with every specific care to reduce the suffering of the mice during the experiments. Mice were euthanized by carbon dioxide asphyxiation.

### Mice

To construct TgV_L_3B4 mice, the V_L_ gene fragment was amplified by polymerase chain reaction (PCR) with cDNA from 3B4 hybridoma as a template, and was used to replace V_L_ gene in plasmid Lκ [27], which contains the promoter region, signal peptide fragment, and J region. A 2kb fragment containing intron enhancer from pBSNB1 and a 10 kb fragment containing the Cκ region, major intron and 3’ enhancer derived from plasmid K1 were then inserted, to generate the κ chain transgene construct pBSCk-2Vk4 (S1 Fig). The 13.5 kb transgene fragment was excised from pBSCk-2Vk4 by *Eco*RI restritction digestion, purified, and microinjected into the pronuclei of fertilized (C57BL/6×CBA) F1 eggs. Injected eggs were transferred to pseudopregnant females to produce transgenic mice. Founder lines were backcrossed with C57BL/6 mice for more than six generations before analyses. The TgV_H/L_3B4 double transgenic mice were produced by crossing TgV_H_3B4I and TgV_L_3B4, and were genotyped by PCR (S1 Fig), with H chain primers described in [26], and L chain primers 5′-CTTCCTGCTAATCAGTGCCTCAG (KLB), and 5′-GTTAGATCTCGAGCTTGGTCC (VKF2). All mice were housed under specific pathogen-free (SPF) conditions, with autoclaved food and water. Eight to twelve week old transgenic mice and age-matched transgene-free littermates were sacrificed for analysis.

### FACS analysis

Single-cell suspensions prepared from the spleen, peritoneal cavity (PEC), lymph nodes (LN), and BM were treated with buffered 0.14 M NH_4_Cl. 5×10^5^ cells were stained with antibodies in PBS containing 2% fetal bovine serum and 0.2% NaN3 for 30 min on ice, followed by washing and filtrating through nylon mesh, and were then fixed in 1% paraformaldehyde or analyzed immediately on a Coulter Epics XL flow cytometer (Beckman coulter). Data were analyzed by using EXPO32 ADC Analysis software (Treestar, San Carlos, CA). Antibodies used in the analyses included anti-B220 (RA3-6B2), anti-IgMa (DS-1), anti-IgMb (AF6-78), anti-κ (187.1), anti-λ (RML-42), anti-CD19 (6D5), anti-IgM (R6-60.2), anti-CD5 (53–7.3), anti-CD21/35 (7G6), anti-CD23 (B3B4), anti-CD24 (M1/69), anti-CD43 (S7), anti-CD1d (1B1), anti-CD138 (281–2). For secondary staining, streptavidin-PE-Cy5, streptavidin-PE, and streptavidin-FITC were used in conjunction with biotinylated antibodies. Antibodies and fluorescence-labeled streptavidin were obtained from BD Biosciences (Mountain View, CA) or BD PharMingen (San Diego, CA). Apoptosis was detected using the Annexin V-FITC Apoptosis Detection Kit (BD PharMingen, San Diego, CA) following standard protocols. FACS-sorting was conducted using a FACS Vantage II (BD Immunocytometry Systems), and sorted cells were re-analyzed by using flow cytometer to confirm cell purity.

### Confocal microscopy

Tissue samples were embedded in OCT, frozen at -80°C, and sectioned at 6 μm thickness. Sections were air-dried, fixed in ice-cold ethanol for 15 min, washed with PBS, and blocked with PBS containing 2% bovine serum albumin. Sections were then stained with anti-B220-PE, anti-IgMa-PE plus anti-MOMA1-FITC (Rat IgG2a, Serotec), or anti-IgM^a^-PE plus anti-IgM^b^-FITC, at room temperature for 30 min, washed, and mounted in 50% glycerol. Images were recorded with a FV1000 confocal microscopic system (Olympus), and were processed with Image Pro Plus 5.1 software.

To measure intracellular Ca^2+^, cells were incubated in DMEM/F12 containing 10 μmol/L Fluo-3 AM (Molecular Probes, Eugene, OR) for 40 min at 37°C, rinsed twice with D-HBSS to remove untreated probes, and scanned under an inverted microscope (model IX81; Olympus) equipped with an Olympus FV-1000 laser scanning confocal imaging system using argon ion laser with an excitation wavelength of 488 nm and a frame rate of 2.5 seconds. Images were analyzed with software Optical FluoView Ver1.5. Fluorescence intensity was normalized to its initial value recorded before 20 μg/ml of actin application.

### Reverse transcription PCR

B220-positive cells were sorted by using magnetic beads-conjugated anti-B220 and a magnetic column (Miltenyi Biotec, Auburn, CA). To the purified B cells Trizol reagent (Promega) was added immediately, and total RNA was prepared according to the manufacturer’s instructions. Approximately 1 μg of total RNA in 20 μl was reverse-transcribed with SuperScript II reverse transcriptase and random primers (Invitrogen Life Technologies). Rag2 was detected using the primers as described [28], with β-actin [29] as a reference control.

### Cell Culture

B220^+^ cells were isolated from the spleen and PEC by using magnetic beads, and cultured at a density of 1×10^5^ cells/ml with RPMI 1640 medium supplemented with 10% fetal bovine serum in U-bottomed 96-well culture plates (Costar) at 37°C. B cell were stimulated with 25 μg /ml lipopolysacharide (LPS), 25 μg /ml anti-IgM, or 15 μg /ml anti-CD40 plus 50 ng/ml interleukin-4 were added as indicated in the different experiments.

### Enzyme-linked immunosorbent assay (ELISA)

Solid-phase ELISA was performed as described previously [30].

### Statistics

Paired *t*-test and one-way ANOVA were used to determine the statistical significance of values between groups. The statistical significance was defined as *P*<0.05.

## Results

### H chain allelic inclusion and receptor editing in B cells of TgV_H_3B4I mice

**Fig 1:**
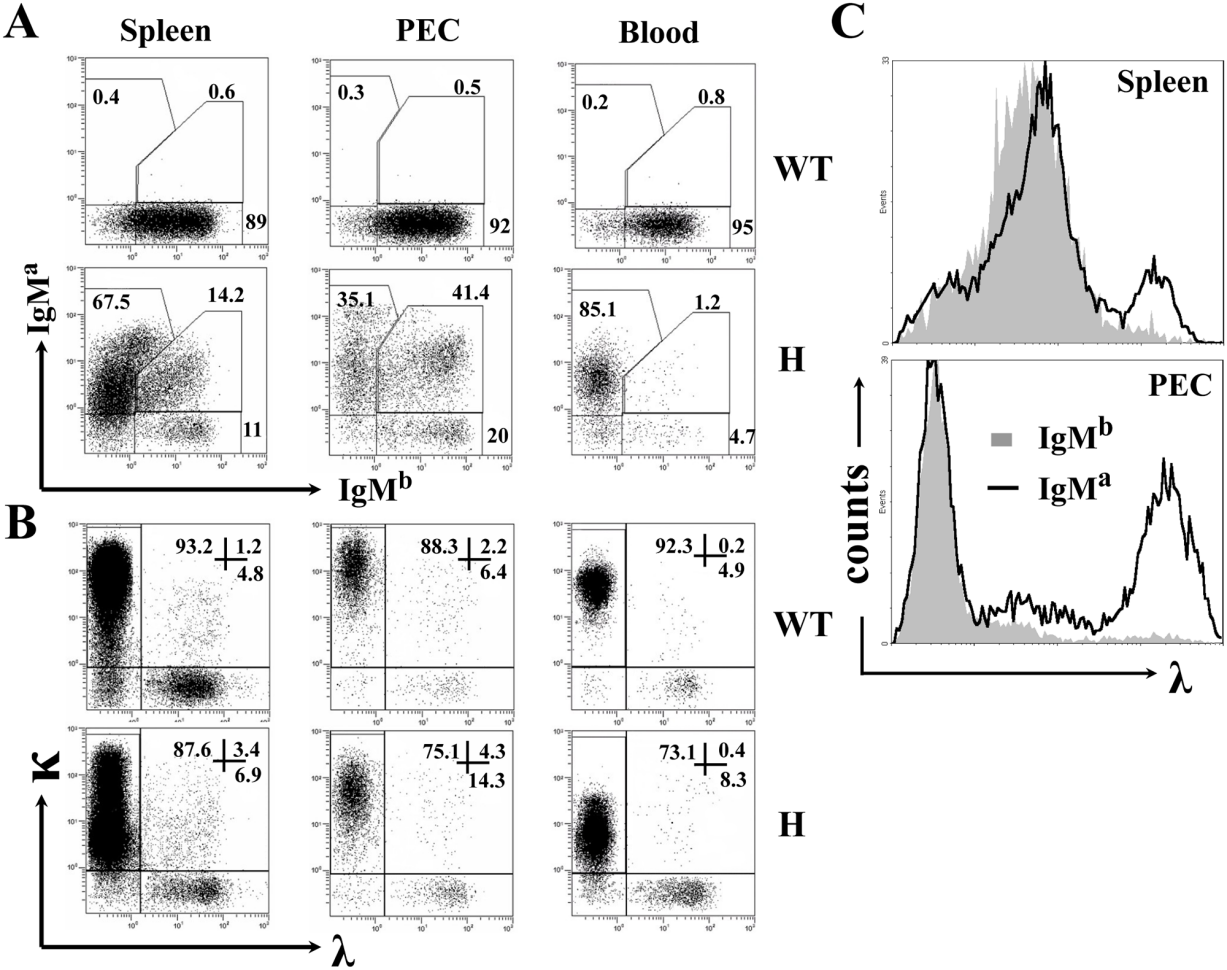
H and L chain expression on peripheral B cells from TgV_H_3B4I. (A) Allelic inclusion of IgH chain on peripheral B cells. Cells from spleen, PEC and peripheral blood of the indicated mice were labeled with anti-IgM^a^, anti-IgM^b^, and anti-CD19, and were analyzed by FACS. CD19^+^ cells were gated on lymphocytes. The numbers indicate the percentage of cells in total CD19^+^ cells within the indicated gates. (B) L chain expression in B cells from TgV_H_3B4I. Cells were stained with anti-κ, anti-λ and anti-CD19 antibodies and were analyzed, as in A. (C) λ-expressing cells are mainly IgM^a+^ in TgV_H_3B4I mice. Histograms show λ chain expression in IgM^b+^ (solid fill) and IgM^a+^ (no fill, including IgM^a+^IgM^b+^ and IgM^a+^IgM^b-^) cells from splenocytes and PEC of TgV_H_3B4I mice. Data are representative of more than eight independent experiments with at least three mice in each group.

TgV_H_3B4I mice carry a μ chain transgene with V_H_ derived from the 3B4 hybridoma that secrete a poly-reactive natural antibody recognizing keratin, actin, myosin and many foreign antigens [26, 30]. To evaluate H chain allelic exclusion in adult TgV_H_3B4I mice, we took advantage of the allotypic difference between the transgenic IgM^a^ and endogenous IgM^b^ of C57BL/6 mice. As shown in Fig 1A (left panels), most splenic B cells from TgV_H_3B4I mice expressed IgM^a^ and a fraction (>14%) expressed both IgM^a^ and IgM^b^ simultaneously, when compared with B cells from C57BL/6 mice. This proportion was more significant in PEC, as more than 40% of the B cells in the PEC of TgV_H_3B4I mice expressed both IgM^a^ and IgM^b^, while B cells expressing IgM^b^ only increased marginally (Fig 1A, middle panels). Interestingly, we found only a few B cells with H chain allelic inclusion (~1%) in the blood (Fig 1A, right panels) and BM (data not shown) of TgV_H_3B4I mice. These results indicated that peripheral B cells underwent H chain allelic inclusion in TgV_H_3B4I mice.

Receptor editing can be elicited by the biased expression of L chains. We found that receptor editing occurred in a significant percentage of each B cell population in TgV_H_3B4I mice, as evidenced by the augmented expression of the λ chain (Fig 1B). Notably, most λ^+^ B cells from PECs of TgV_H_3B4I mice were IgM^a+^ that constituted the autoreactive BCR (Fig 1C, lower panel), suggesting that receptor editing might be involved in the development of autoreactive B cells in TgV_H_3B4I mice.

### B cells with H chain allelic inclusion develop preferentially into MZ and B-1a cells in TgV_H_3B4I mice

**Fig 2:**
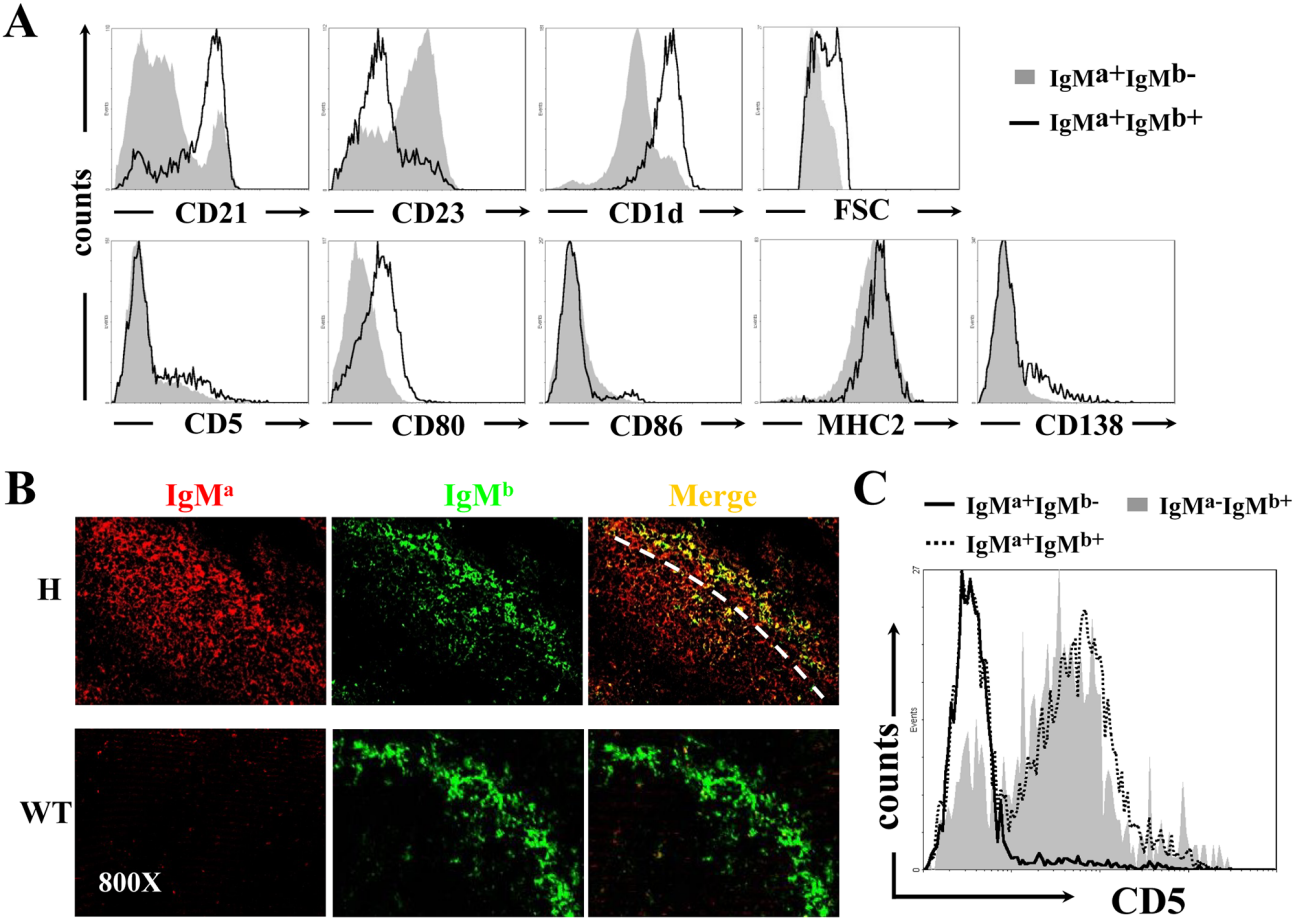
Accumulation of B cells with allelic inclusion in the MZ of spleen and their differentiation into B-1a subset in PEC of TgV_H_3B4I mice. (A) Phenotypic analysis of IgM^a+^IgM^b-^ (solid fill) and IgM^a+^IgM^b+^ (no fill) B cells for the indicated cell surface markers by flow cytometry from splenocytes of TgV_H_3B4I mice. (B) IgM^a+^IgM^b+^ B cells localized in the MZ of spleen of TgV_H_3B4I. Analysis of frozen spleen sections from TgV_H_3B4I mice, stained with anti-IgM^a^ (red) and anti-IgM^b^ (green), using confocal microscopy. (C) CD5 was expressed predominantly on IgM^a-^IgM^b+^ and allelic included IgM^a+^IgM^b+^ B cells. Representative histograms show the surface expression of CD5 on IgM^a-^IgM^b+^ (solid fill), IgM^a+^IgM^b-^ (solid line) and IgM^a+^IgM^b+^ (dotted line) B cells of PEC from TgV_H_3B4I mice. Data are representative of more than five independent experiments with three mice per group.

To investigate the effects of H chain allelic inclusion on B cell development in TgV_H_3B4I mice, we examined the phenotypes of IgM^a+^ B cells, which could be divided into IgM^a+^IgM^b-^ (allelic excluded) and IgM^a+^IgM^b+^ (allelic included) subpopulations in the spleen and PEC. In the spleen, IgM^a+^IgM^b+^ B cells expressed high level of CD21 and CD1d, but lower level of CD23 (Fig 2A, upper panels), a phenotype identical to that of marginal zone (MZ) B cells. In contrast, most IgM^a+^IgM^b-^ B cells had a follicular (Fo) B cell-like phenotype of CD21^low^CD23^high^CD1d^low^. Histological analysis using anti-IgM^a^ and anti-IgM^b^ confirmed that B cells with allelic inclusion were mainly located at the MZ of the spleen in TgV_H_3B4I mice when compared with C57BL/6 mice (Fig 2B). Thus, B cells with H chain allelic inclusion, which were most likely poly-reactive, mainly developed into MZ B cells in the spleen of TgV_H_3B4I mice. Moreover, allelic included B cells expressed slightly higher level of activation markers, such as CD80 and MHC-II (Fig 2A, lower panels), and had larger cellular size than IgM^a^ single positive B cells (Fig 2A), suggesting BCR engagement or activation [31, 32].

In the PEC of TgV_H_3B4I mice, almost all of the IgM^a+^IgM^b-^ B cells were CD5^-^ (Fig 2C), indicating that they belonged to B-2 and B-1b subsets. In contrast, the majority of IgM^a+^IgM^b+^ B cells were CD5^+^ B-1a cells (Fig 2C). These results suggested that B cells with allelic inclusion preferentially developed into B-1a cells in PECs of TgV_H_3B4I mice.

### Analysis of B cell development in the BM of TgV_H_3B4I and TgV_H/L_3B4 mice

**Fig 3:**
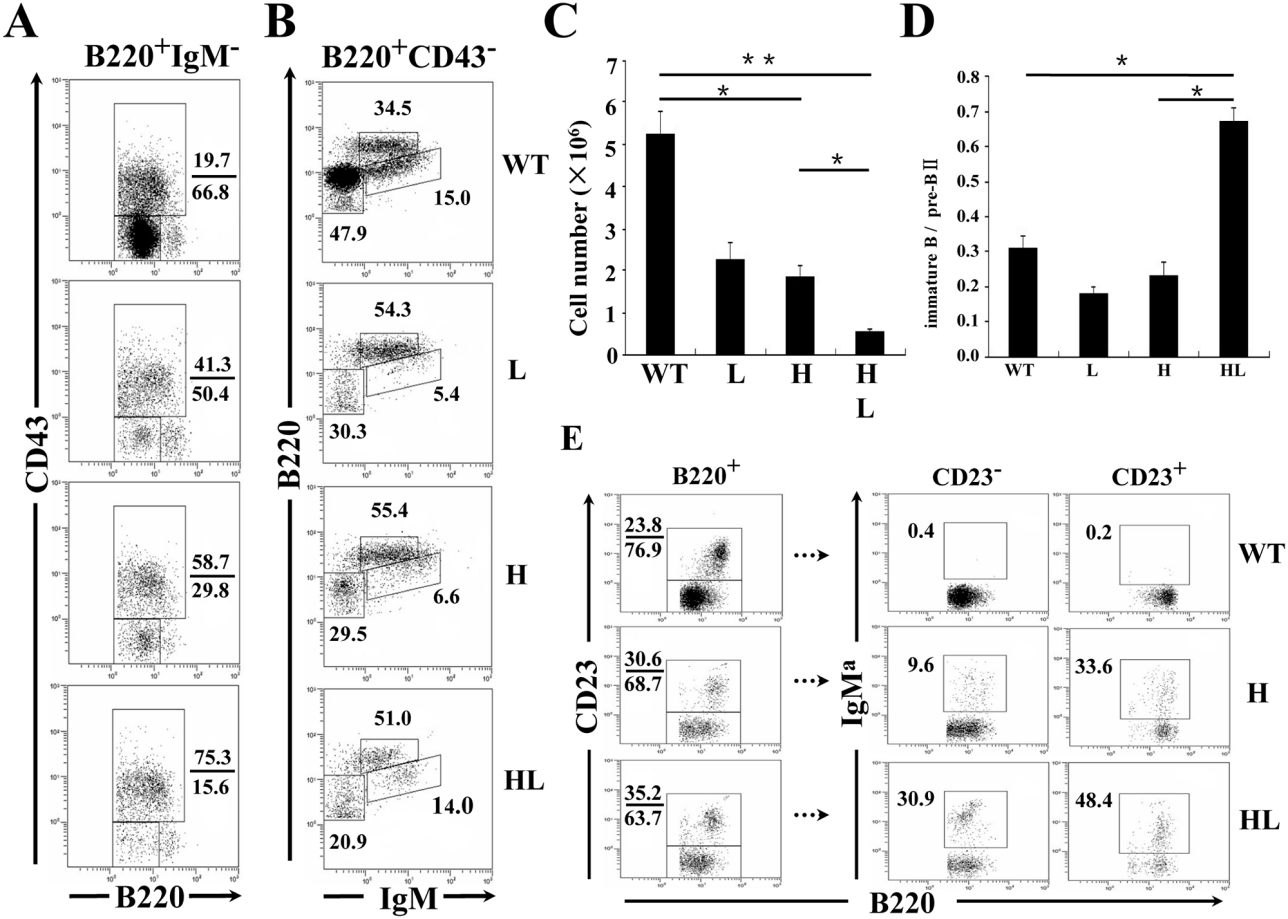
Assessment of B cell development in the BM of transgenic mice. BM cells from the indicated mouse strains were stained with anti-B220, anti-CD43, and anti-IgM, and were analyzed by FACS. (A) The gates in panels represent Hardy fractions A-D (proB to preBII), and can be divided into fraction A-C (proB to preBI, upper rectangle) and fraction D (preBII, lower rectangle) by the expression of CD43. The numbers indicate the percentage of cells within the gates. (B) Hardy fractions D (preBII, lower left panels), F (circulating mature B cells, upper middle panels) and E (immature B cells, lower right panels) of the indicated mice. (C) Decreased absolute numbers of total BM B cells in transgenic mice. Numbers of B220^+^ cells were calculated and shown. (D) Numbers of preBII and immature B cells in B were calculated, and the ratios of immature cells to preBII cells were calculated as shown. Error bars represent means ± SD, *P<0.05, **P<0.01, n = 5. (E) FACS analysis of BM B cells from the indicated transgenic mice. CD23^-^ (newly formed) and CD23^+^ (mature) B cells in the B220^+^ gate are shown in the boxes. Data are representative of more than five independent experiments.

Our data showed that transgenic expression of 3B4 H chain resulted in significant receptor editing and H chain allelic inclusion. Next, we investigated the BM development of original 3B4 B cells. The 3B4 κ chain transgenic mouse line TgV_L_3B4 was established and was bred with TgV_H_3B4I to generate TgV_H/L_3B4 mice. In the BM, where clonal deletion and receptor editing of autoreactive B cells mainly takes place [33, 34], both the percentage and absolute number of total B cells were significantly reduced in different transgenic lines compared to C57BL/6 mice (Fig 3A–3C), most likely due to the early expression of the Ig transgenes [35, 36]. Indeed, this decrease was mainly attributable to the reduction of early B cells, because IgM^-^ B cell precursors decreased most significantly (Fig 3A–3C and data not shown). There was also a severe reduction of preBII cells (IgM^-^CD43^-^) (Fig 3A) in TgV_H/L_3B4 mice, as evidenced by the highest ratio of pro/preBI (IgM^-^CD43^+^) to preBII cells in this line (Fig 3D). This might explain the observed significant reduction in total B cells number in the BM of TgV_H/L_3B4 mice (Fig 3C), because the preBII stage is critical to the proliferation of BM B cells.

B220^int^IgM^high^CD43^-^ immature B cells were comparable between TgV_H/L_3B4 (~14%) and control littermates (~15%), (Fig 3B). In contrast, the proportion of immature B cells decreased significantly in TgV_H_3B4I and TgV_L_3B4 single transgenic mice (Fig 3B), consistent with a developmental block at the immature stage in B cells expressing single transgenic L or H chains. A higher ratio of immature B to preBII was noticed in TgV_H/L_3B4 mice, suggesting that more immature B cells were generated in this line compared with the H or L chain single transgenic mice (Fig 3D). Similarly, in the CD23^-^ immature B cell stage, the percentage of IgM^a+^ cells was much higher in TgV_H/L_3B4 than in TgV_H_3B4I (Fig 3E). These results suggested that B cells expressing the 3B4 natural poly-reactive BCR could be positively selected in the BM.

### Higher allelic exclusion efficiency in the B cells from TgV_H/L_3B4 mice

**Fig 4:**
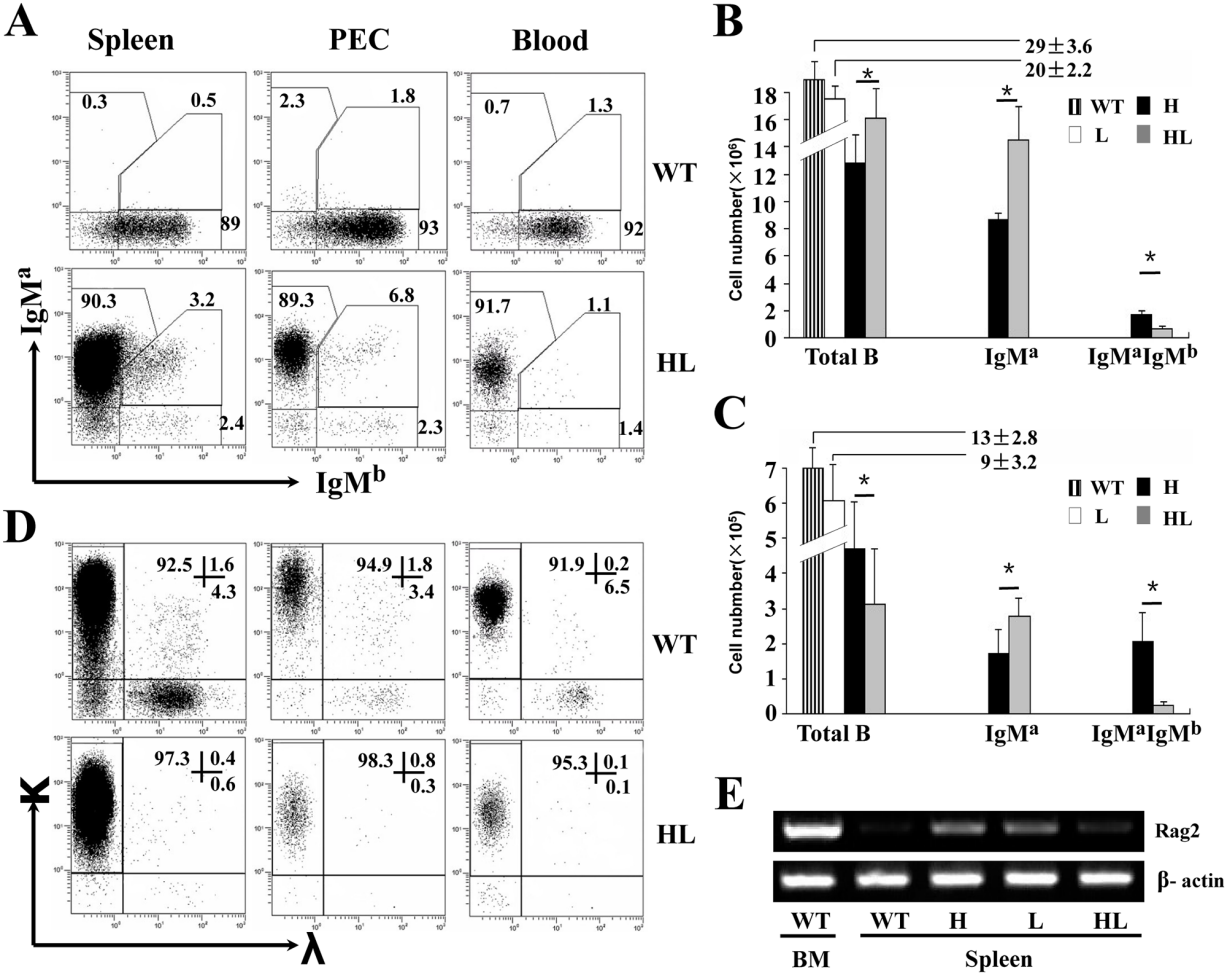
High allelic exclusion of H and L chains in peripheral B cells of TgV_H/L_3B4 mice. (A) Expression of H chains in the peripheral B cells. Cells from spleen, PEC and peripheral blood of TgV_H/L_3B4 mice were stained and analyzed as in Fig 1A. (B-C) Bar graph shows the absolute numbers of total, IgM^a+^IgM^b-^ and IgM^a+^IgM^b+^ B cells as gated in A in the spleen (B) and PEC (C) of the indicated mice. Error bars show mean ±SD, *P<0.05. (D) L chain expression in B cells from TgV_H/L_3B4 mice. Cells were stained with anti-κ, anti-λ and anti-CD19, and were analyzed by FACS. (E) RT-PCR analysis of Rag2 mRNA expression in B cells of spleen and BM from transgenic mice and littermates. Data are representatives of five independent experiments.

Over 90% of splenic, PEC and blood B cells in TgV_H/L_3B4 mice expressed IgM^a^ alone, while less than 3% expressed IgM^b^ (Fig 4A). Fewer number of B cells expressed both IgM^a^ and IgM^b^ in the spleen and PEC in TgV_H/L_3B4 mice, in contrast to TgV_H_3B4I mice in which more B cells underwent allelic inclusion in the spleen and PEC (Fig 4B and 4C). This suggested that allelic inclusion was suppressed in TgV_H/L_3B4 mice compared to the H chain transgenic mice. Consistently, the number of IgD^+^ B cells was more than three folds lower in spleen and PEC of TgV_H/L_3B4 than that of TgV_H_3B4I mice (S2 Fig).

Lower percentage of B cells expressed λ L chain in the spleen, PEC and blood in TgV_H/L_3B4 mice compared to controls (Fig 4D). The λ^+^ B cells were even lower than that in TgV_L_3B4 mice (data not shown), suggesting reduced λ chain inclusion and receptor editing. Consistently, RAG2 [37] mRNA transcript level increased in the splenic B cells of TgV_H_3B4I and TgV_L_3B4 mice, but was comparable to wild type in TgV_H/L_3B4 mice (Fig 4E). These results indicated that receptor editing and allelic inclusion were induced in B cells of single H or L chain transgenic mice, but were suppressed in B cells overexpressing the intact 3B4 BCR.

### Evaluation of B cell development in the periphery of TgV_H/L_3B4 mice

**Fig 5:**
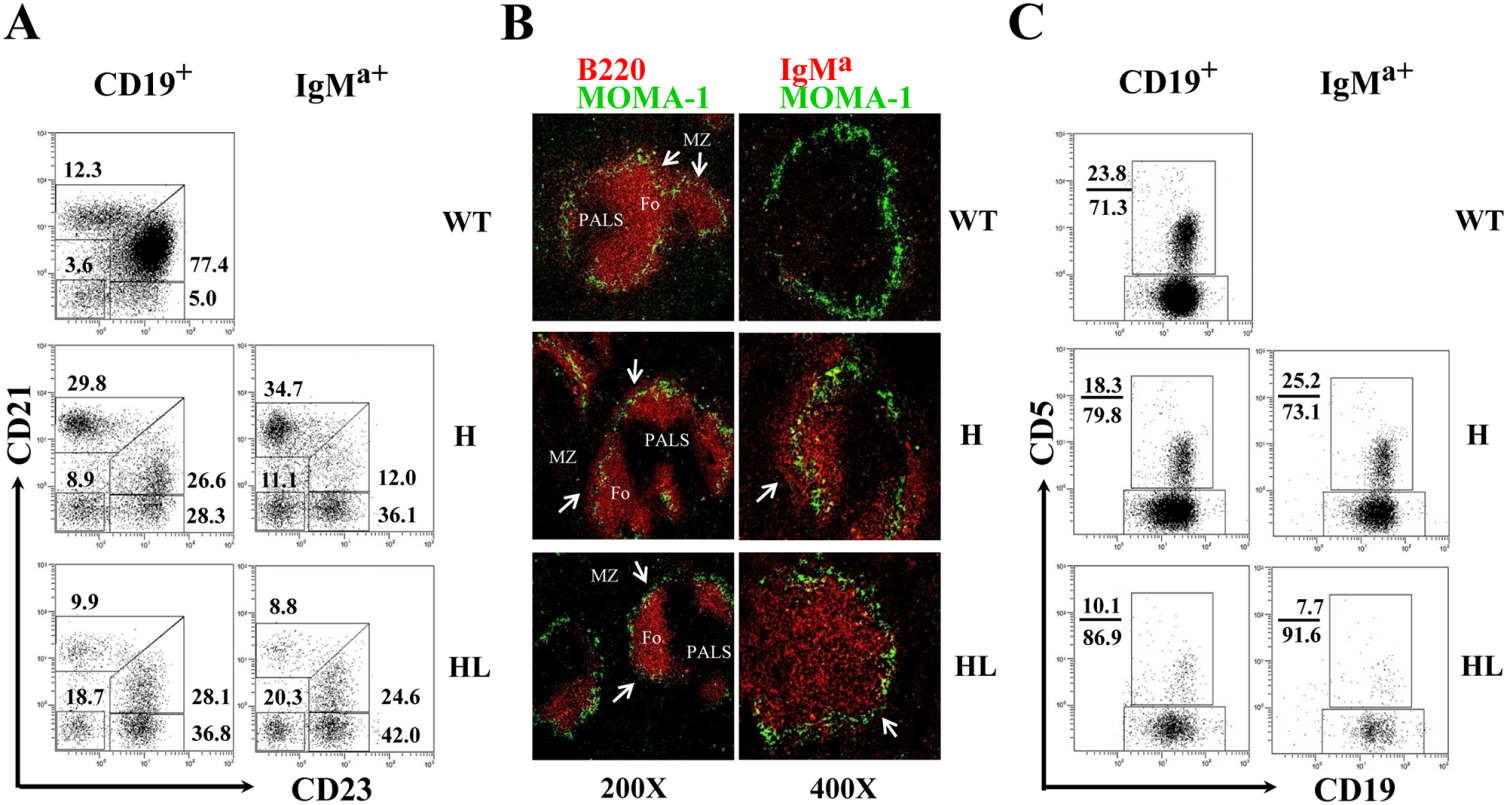
Analysis of B cells development in spleen and PEC of transgenic mice. (A) The phenotype of B cells in the spleen of TgV_H_3B4I and TgV_H/L_3B4 mice. CD19^+^ or IgM^a+^ splenic B cells from indicated mice were analyzed for the expression of CD21 and CD23 by FACS. Numbers next to each gate indicate the percentage of cells in that gate in total CD19^+^ or IgM^a+^ cells. At least 7 mice from each genotype were analyzed. (B) Spleen sections from the indicated mice were stained with anti-MOMA1-FITC and anti-B220-Biotin (left panels) or anti-IgM^a^-biotin (right panel) followed by streptavidin-Cy3, and acquired using fluorescence microscopy. Follicular (Fo) areas around PALS are shown and MZ is indicated with arrow. The original magnitude was ×200 or ×400 as indicated. (C) CD19^+^ or IgM^a+^ B cells in PEC of indicated mice were evaluated for the expression of CD5 by FACS. At least 5 mice from each genotype were analyzed. Numbers next to each gate indicate the percentage of cells in that gate in total CD19^+^ or IgM^a+^ cells.

The percentage of MZ B cells (CD19^+^CD21^high^CD23^low^) increased significantly in TgV_H_3B4I mice, but this population decreased in TgV_H/L_3B4 compared to WT littermates (Fig 5A). Consistently, immunofluorescence analysis of spleen sections showed that compared to TgV_H_3B4I mice, with normal or somewhat enlarged MZ B cell area, in TgV_H/L_3B4 mice the MZ B cell area was significantly reduced (Fig 5B). However, Fo B cells were significantly reduced in both the mouse lines, likely due to the reduced BM cellular export (S3 Fig). We did not observe a significant difference in the apoptosis of Fo B cells and MZ B cells of TgV_H_3B4I and TgV_H/L_3B4 mice (S4 Fig), suggesting that the differential development of splenic B cells in TgV_H_3B4 and TgV_H/L_3B4 mice was most likely due to altered positive selection. Moreover, a majority of splenic B cells from TgV_H_3B4I and TgV_H/L_3B4 mice developed into a particular subset with a phenotype of CD21^-^CD23^high^CD24^low^, distinct from the typical T2 phenotype of CD21^int-high^CD23^high^CD24^high^ (Fig 5A and data not shown). Most of these cells termed as T2’, were IgM^a+^ in both TgV_H_3B4I and TgV_H/L_3B4 (Fig 5A and 5B, right panels). Similarly, B cells in the blood of TgV_H_3B4I and TgV_H/L_3B4 were also mainly composed of T2’ cells, which were distinct from circulating B cells of the controls (data not shown). These results indicated that 3B4 poly-reactive B cells developed into special B cell subsets.

In the PEC of TgV_H_3B4I mice, the percentage of B-1 cells (CD19^+^Mac1^+^) increased but CD5^+^ B-1a cells decreased (data not shown). However, within the IgM^a+^ population of TgV_H_3B4I mice, the percentage of CD5^+^ B-1a cells increased notably (Fig 5C, middle panels). As discussed previously, majority of these CD5^+^IgM^a+^ cells were B cells with H chain allelic inclusion (Fig 2C). In TgV_H/L_3B4 mice, while B-2 cells (CD19^+^Mac1^-^) were comparable to the littermate controls, more than 80% of B-1 cells were B-1b cells (CD19^+^Mac1^+^CD5^-^) (data not shown). IgM^a+^CD5^+^ cells decreased more significantly (to about 10% of B cells) in TgV_H/L_3B4 mice when compared to TgV_H_3B4I mice (Fig 5C). The apparent reduction of B cell subsets in the PEC of TgV_H_3B4I or TgV_H/L_3B4 mice could be the result of lower B cell production in the BM or due to their negative selection in the PEC. These data were further supported by the lower number of B-1a and B-1b cells in PEC of TgV_H/L_3B4 compared with TgV_H_3B4I mice (S3B Fig), in which B cells possibly escaped negative selection through allelic inclusion. Consistent with this opinion, the ratio of apoptotic CD5^+^ and CD5^-^ B cells was significantly higher in PEC of TgV_H/L_3B4 mice (21%/35%) compared with the littermate controls (5%/16%). However, this ratio remained low in TgV_H_3B4I mice (8%/25%) (S4B Fig).

### B cells can secrete poly-reactive autoantibodies independent of H chain allelic inclusion

**Fig 6:**
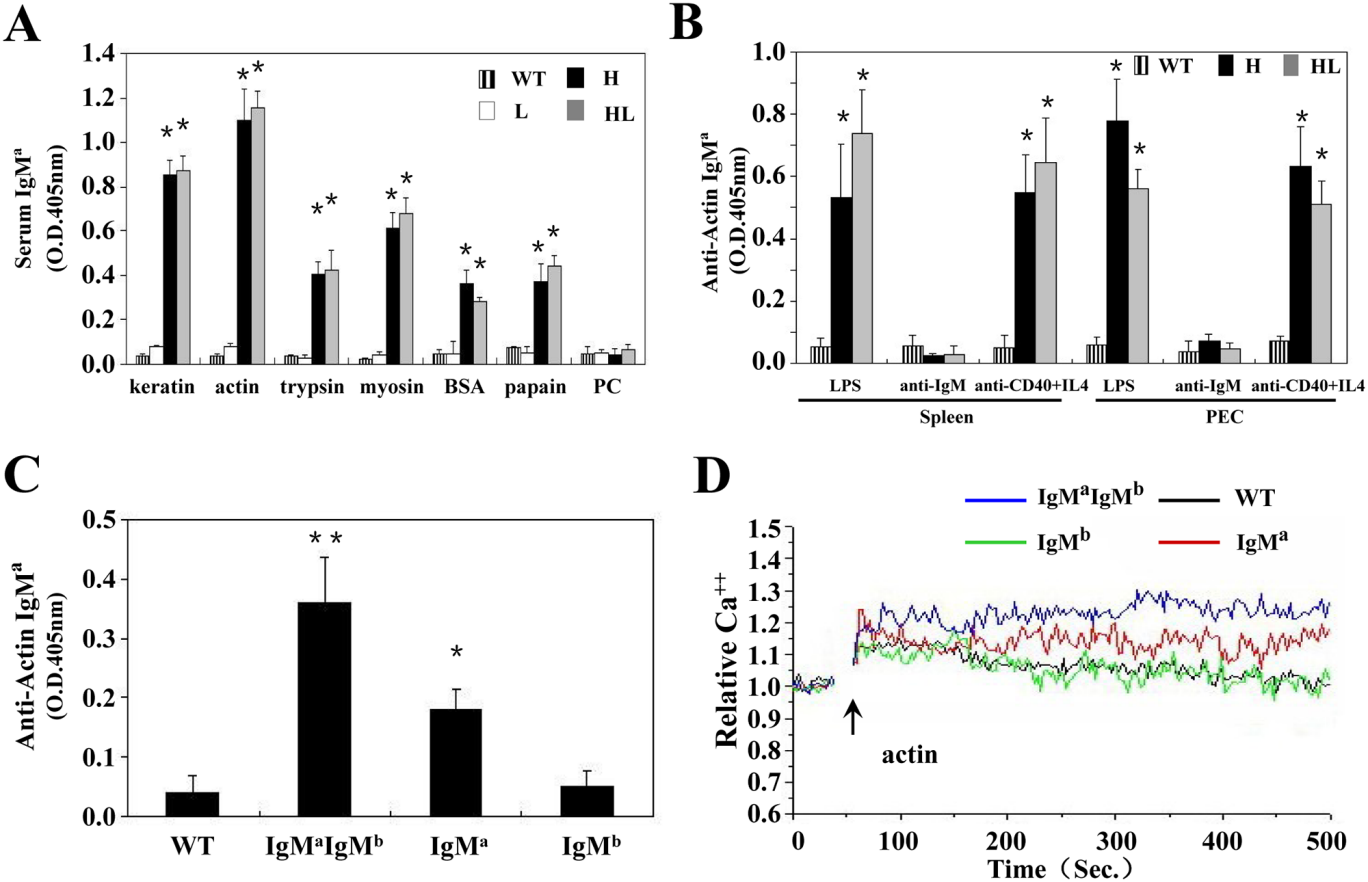
Antibody production by allelic included B cells in TgV_H_3B4I. (A) ELISA. Sera from at least 5 mice from each genotype were collected, and analyzed with ELISA using standard protocols. (B) In vitro secretion of autoantibodies by B cells. Purified B cells from spleen and PEC of indicated mice were treated with LPS, anti-IgM and anti-CD40+IL-4 for 3 days. Supernatants were collected and the concentration of secreted antibodies was assessed by ELISA. (C) B cells were sorted from spleens of TgV_H_3B4I mice and were treated with LPS, and antibody production was tested as in B. Error bars show means ± SD. *P<0.05, **P<0.01, n = 5. (D) Calcium mobilization analysis. Sorted splenic B cells from TgV_H_3B4 mice and littermate controls were stained with Fluo3. Cells were stimulation with actin (20 μg/ml) and analyzed by FACS.

We next characterized the poly-reactivity of antibodies produced by cultured B cells from mice with allelic inclusion (TgV_H_3B4I) or not (TgV_H/L_3B4) by ELISA. B cells in both of TgV_H_3B4I and TgV_H/L_3B4 mice produced high titer of IgM^a^ in the serum, with antigenic reactivity similar to that of 3B4 hybridoma (Fig 6A), suggesting that the 3B4 H chain determined the poly-reactivity of these B cells in conjunction with 3B4 or allelic included L chains. Moreover, B cells from both spleen and PEC of TgV_H_3B4I and TgV_H/L_3B4 mice produced high concentration of autoantibodies upon in-vitro stimulation with LPS or anti-CD40 (Fig 6B).

IgM^a+b-^, IgM^a+b+^, and IgM^a-b+^ B cells, which represented H chain allelic excluded, included, and wild type B cells, respectively, were sorted from splenocytes of TgV_H_3B4I mice, and tested for autoantibody production. IgM^a+^IgM^b+^ cells secreted anti-actin autoantibodies at higher titers than IgM^a+b-^ cells upon LPS stimulation (Fig 6C), suggesting that autoantibody production was significantly contributed by the B cells that underwent allelic inclusion in the spleen of TgV_H_3B4I mice. Consistently, IgM^a+^IgM^b+^ cells showed a relatively sustained and elevated calcium flux than IgM^a+b-^ B cells when stimulated with actin (Fig 6D). These effects were not observed in the IgM^a-b+^ cells or wild type control. These data suggested that B cells in TgV_H_3B4I mice with H chain allelic inclusion had a considerable ability to produce autoantibodies.

## Discussion

In the present study, we compared B cells development in mice bearing H chain, L chain or H+L chains from 3B4 hybridoma, which produces poly-reactive antibodies recognizing autoantigens as well. Our results suggest that in TgV_H_3B4I mice, B cell development is accompanied by allelic inclusion, which results from receptor editing, leading to the maturation of B cells secreting poly-reactive antibodies. B cells in TgV_H_3B4I might undergo negative selection at the first tolerance checkpoint (the immature B cells stage), as we observed a remarkable decrease in immature B cells in TgV_H_3B4I mice. However, poly-reactive B cells entering the second tolerance checkpoint at the transitional B cell stage might be positively selected after receptor editing results in allelic inclusion. Allelic inclusion might rescue poly-reactive B cells from negative selection by creating dual receptor expression on their surface. Moreover, H chain allelic inclusion may serve as a special mechanism of positive selection to permit the survival of autoreactive B cells in the periphery [38]. We also found that receptor editing occurred significantly in B cells of TgV_H_3B4I mice, as evidenced by the augmented expression of the λ chain (Fig 1B). Sirac has reported that light chain exclusion prevalent in normal B cells is neither tightly ensured by a stringent cell selection process nor absolutely required for normal B cell function [39]. Similarly, B cells of TgV_H_3B4I mice can further differentiate into mature B cell compartments and produce poly-reactive antibodies. These findings in TgV_H_3B4I mice are consistent with previous reports [23] showing that autoreactive antibodies in the serum of normal mice and humans may be the “fellow travelers” in B cells with allelic inclusion.

When the original 3B4 L chain was introduced into TgV_H_3B4I mice, receptor editing was severely suppressed. Few, if any, λ^+^ B cells were found in BM or the periphery in TgV_H/L_3B4 mice. However, TgV_H/L_3B4 mice still produced high titers of poly-reactive antibodies in the serum, indicating that the 3B4 natural poly-reactive B cells could develop into mature B cells without receptor editing. The low avidity of the 3B4 natural autoantibody could potentially help these B cells to escape negative selection mechanisms in both the BM and periphery. Meanwhile, 3B4 poly-reactive B cells were also positively selected through specific recognition of certain autoantigens [40]. Our data suggest that in TgV_H_3B4I mice, the incorporation of secondary non-original L chains at early stages of B cell development would change the original avidity and/or specificity of the BCR, and subsequently trigger receptor editing in the BM and periphery. Alternately, in TgV_H/L_3B4 mice, B cells with intact 3B4 BCR would directly differentiate into mature B cell subsets without further receptor editing, and secrete poly-reactive autoantibodies. Therefore, receptor editing plays a minor role in the development of 3B4 poly-reactive B cells.

We observed that a majority of 3B4 B cells developed into a special B cells subset that we referred to as T2’ cells in the spleen of TgV_H/L_3B4 mice. These B cells were sensitive to the BCR crosslink-induced apoptosis [41] (Fig 6B and data not shown), suggesting that they underwent negative selection before maturing into poly-reactive antibody secreting B cells. Although 3B4 hybridoma was originally developed from splenocytes [30], we could not define the phenotype of original 3B4 B cells. However, the T2’ phenotype could result from the overexpression of 3B4 BCR on the B cell surface and consequently increased tonic signaling. In the PEC, the 3B4 B cells exhibited a phenotype reminiscent of CD5^-^ “B-1b” cells, a B cells subset providing protection from pathogens [42]. This could be a result of positive selection, depending on BCR signal strength [43], as reported previously [44–46]. A similar situation might also exist in TgV_H_3B4I mice, in which the frequency of B cells with H chain allelic inclusion is high in the MZ and B-1a cell compartments.

In conclusion, our findings suggest that receptor editing plays a minor role in the positive selection of B cells expressing natural poly-reactive BCRs, and these B cells can be positively selected through heavy chain allelic inclusion to retain their poly-reactivity in the periphery.

## Supporting Information

S1 Fig Generation of V_H/L_3B4 transgenic mouse. (A) The structure of the 3B4V_L_ transgene with exons and introns represented by boxes and lines respectively. (B) PCR analysis of the genome DNA from tails of the offspring of TgV_H_ 3B4I and TgV_L_3B4 mice. Each genotype is indicated.
(TIF)

S2 Fig IgM and IgD expression in transgenic mice. Cells from spleen, peritoneal cavity and blood of indicated mice were labeled with a combination of anti-IgM, anti-IgD, and anti-CD19 mAbs and analyzed by flow cytometry with the same strategy as Fig 1A. Values indicate the percentage of events in each quadrant relative to the total number of CD19^+^ cells. Data are representative of more than eight independent experiments.
(TIF)

S3 Fig Absolute B cells number of different B cell compartments. (A) Comparison between splenic B cell subsets from indicated mice. The bar chart summarize the absolute numbers calculated from dot plot data represented mainly in Fig 5A. (B) Comparison between B cell subsets of peritoneal cavity from the indicated mice. The bar charts summarize the absolute cell numbers calculated from dot plot data represented in Fig 5C and data not shown.
(TIF)

S4 Fig Apoptosis analysis of peripheral B cells subsets in transgenic mice. (A) CD21^high^IgM^high^ (mainly MZ B), CD21^low^IgM^low^ (mainly Fo B) and B220^+^IgM^a+^ B cells in the spleen of indicated mice were evaluated for the apoptosis through a combination staining of PI and AnnexinV and then analyzed by flow cytometry. The percentage of live (lower left) and early apoptotic (lower right) cells in each gates are indicated. (B) CD19^+^CD5^-^ (B-1b and B-2) and CD19^+^CD5^+^ (B-1a) B cells in the peritoneal cavity of indicated mice were evaluated for the apoptosis as described above. Data represented three independent experiments with at least three mice in each genotype.
(TIF)
