# Supplementary figures and images for: Positive Selection of Natural Poly-Reactive B Cells in the Periphery Occurs Independent of Heavy Chain Allelic Inclusion
Source: PLoS One. 2015 May 19;10(5):e0125747. doi: 10.1371/journal.pone.0125747 (PMC4437983; doi:10.1371/journal.pone.0125747)

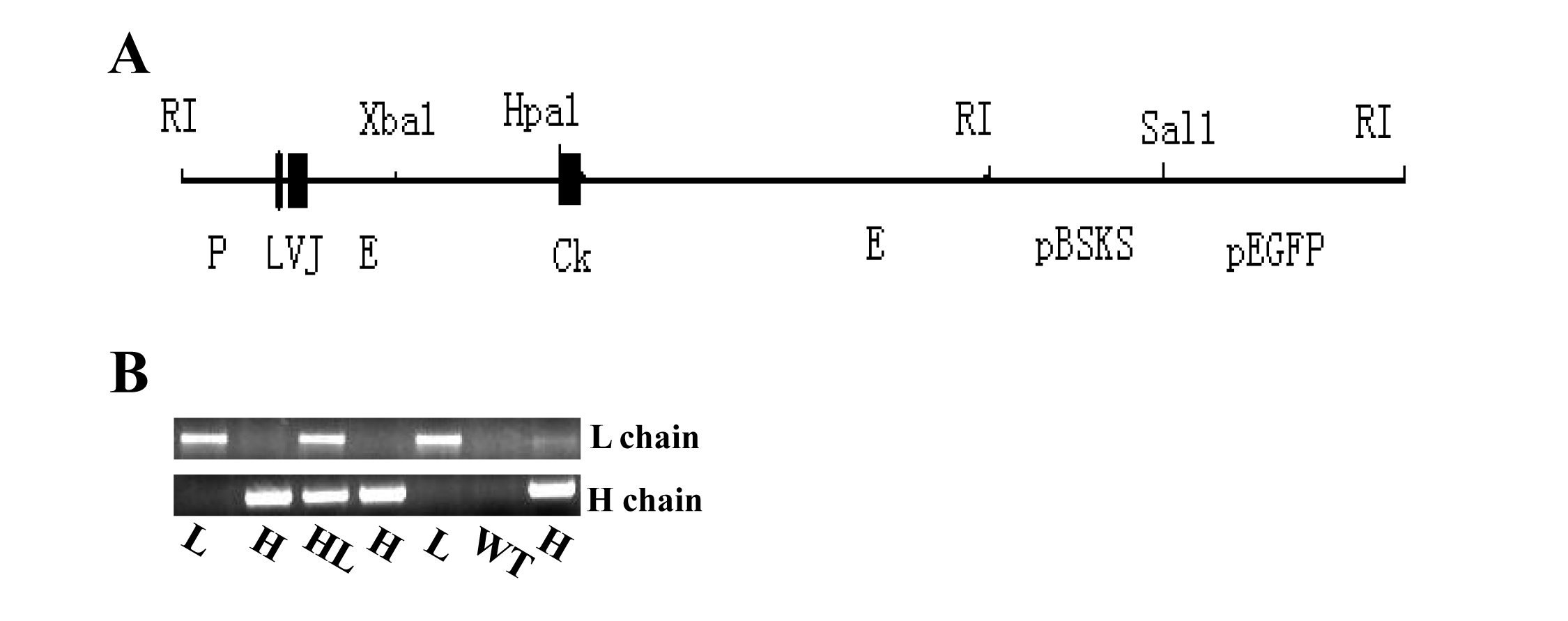

Supplement: S1 Fig — (A) The structure of the 3B4VL transgene with exons and introns represented by boxes and lines respectively. (B) PCR analysis of the genome DNA from tails of the offspring of TgVH 3B4I and TgVL3B4 mice. Each genotype is indicated. (TIF) [file pone.0125747.s001.tif]

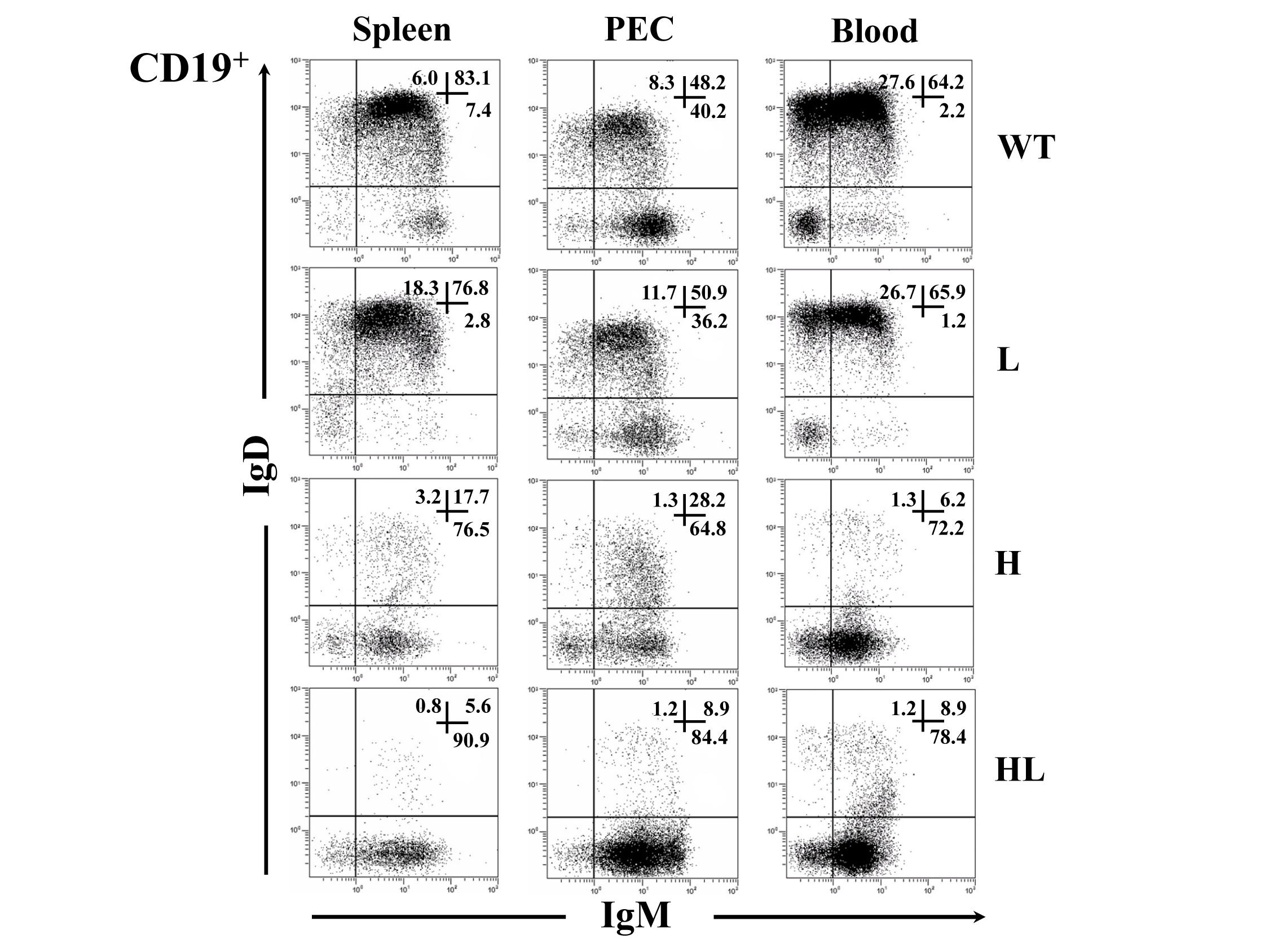

Supplement: S2 Fig — Cells from spleen, peritoneal cavity and blood of indicated mice were labeled with a combination of anti-IgM, anti-IgD, and anti-CD19 mAbs and analyzed by flow cytometry with the same strategy as Fig 1A. Values indicate the percentage of events in each quadrant relative to the total number of CD19+ cells. Data are representative of more than eight independent experiments. (TIF) [file pone.0125747.s002.tif]

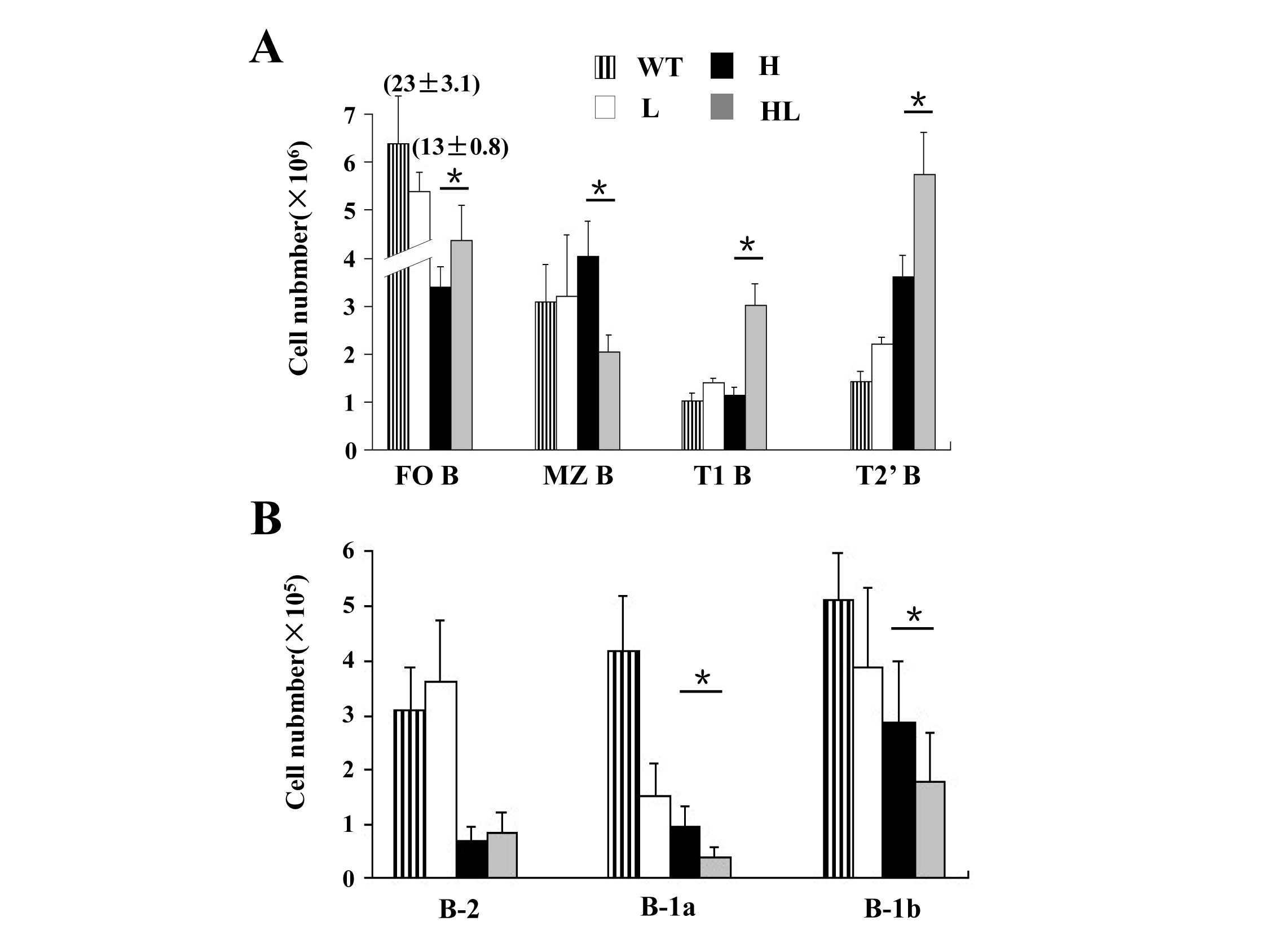

Supplement: S3 Fig — (A) Comparison between splenic B cell subsets from indicated mice. The bar chart summarize the absolute numbers calculated from dot plot data represented mainly in Fig 5A. (B) Comparison between B cell subsets of peritoneal cavity from the indicated mice. The bar charts summarize the absolute cell numbers calculated from dot plot data represented in Fig 5C and data not shown. (TIF) [file pone.0125747.s003.tif]

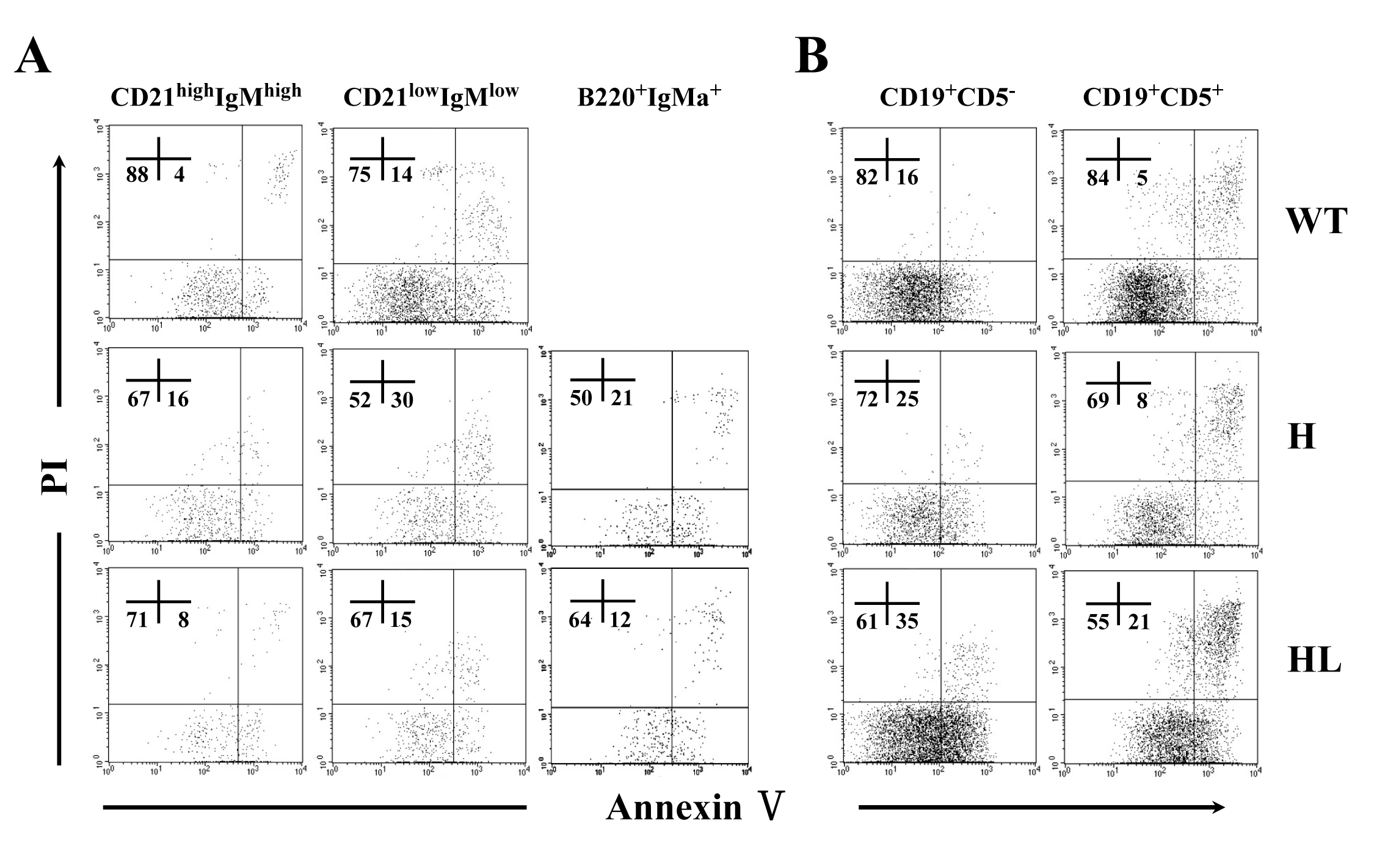

Supplement: S4 Fig — (A) CD21highIgMhigh (mainly MZ B), CD21lowIgMlow (mainly Fo B) and B220+IgMa+ B cells in the spleen of indicated mice were evaluated for the apoptosis through a combination staining of PI and AnnexinV and then analyzed by flow cytometry. The percentage of live (lower left) and early apoptotic (lower right) cells in each gates are indicated. (B) CD19+CD5- (B-1b and B-2) and CD19+CD5+ (B-1a) B cells in the peritoneal cavity of indicated mice were evaluated for the apoptosis as described above. Data represented three independent experiments with at least three mice in each genotype. (TIF) [file pone.0125747.s004.tif]
